# Salvage Reirradiation with Proton Beam Therapy for Locoregionally Recurrent Non-Small Cell Lung Cancer

**DOI:** 10.3390/cancers16213587

**Published:** 2024-10-24

**Authors:** Matthew S. Ning, Abigael Odwuor, Joe Y. Chang, Saumil Gandhi, Zhongxing Liao, Steven H. Lin, Aileen Chen, James W. Welsh, Quynh-Nhu Nguyen, Michael S. O’Reilly, Stephen G. Chun, Julianna Bronk, David Qian, Percy Lee

**Affiliations:** 1 Department of Thoracic Radiation Oncology, The University of Texas MD Anderson Cancer Center, Houston, TX 77030, USA; abodwuor@gmail.com (A.O.);; 2 Department of Radiation Oncology, City of Hope Orange County, Lennar Foundation Cancer Center, Irvine, CA 92618, USA; percylee@coh.org

**Keywords:** reirradiation, proton beam therapy, non-small cell lung cancer

## Abstract

Background/Objectives: This retrospective study evaluates outcomes of 66 patients who underwent reirradiation (re-RT) with proton beam therapy (PBT) for recurrent non-small cell lung cancer. Methods: Toxicity was scored via the CTCAE v5.0, and outcomes estimated using the Kaplan–Meier method, with associations evaluated via Cox proportional hazards and logistic regression analyses. Results: Patients were treated to a median re-RT prescription of 66 Gy/33 fxs (BED10 = 79 Gy; IQR: 71–84 Gy) at an interval of 1.4 years from prior RT. Half (50%) received concurrent chemotherapy. At 14 months follow-up, the median OS and PFS were 5 months (95%CI: 13–17) and 12.5 months (95%CI: 10–15), respectively. On multivariable analysis, a higher RT dose (BED10 > 70 Gy) [HR0.37; 95%CI: 0.20–0.68, *p* = 0.001] and concurrent chemotherapy (HR0.48; 95%CI: 0.28–0.81, *p* = 0.007) were associated with improved PFS, while treatment site overlap was adversely associated (HR1.78; 95%CI: 1.05–3.02, *p* = 0.031). The median PFS for definitive RT with concurrent chemotherapy (*n* = 28), definitive RT alone (BED10 > 70 Gy) [*n* = 22], and lower prescription RT (BED10 < 70 Gy) [*n* = 16] was 15.5 months (95%CI: 7.3–23.7), 14.1 months (95%CI: 10.9–17.3), and 3.3 months (95%CI: 0–12.3), respectively (log-rank, *p* = 0.006), with corresponding 2-year estimates of 37% (±9), 18% (±8), and 12.5% (±8), respectively. The incidence of Grade 3+ toxicity was 10.5% (6% pulmonary; 3% esophageal; and 1.5% skin), including one Grade 4 bronchopulmonary hemorrhage but no Grade 5 events. Cases with central site overlap had higher composite Dmax to the esophagus (median 87 Gy [IQR:77–90]), great vessels (median 120 Gy [IQR:110–138]), and proximal bronchial tree (median 120 Gy [IQR:110–138]) as compared to other cases (*p* ≤ 0.001 for all). However, no significant associations were identified with Grade 3+ events. Conclusions: Thoracic re-RT with PBT is an option for recurrent NSCLC with acceptable outcomes and toxicity for select patients. When feasible, higher prescription doses (BED10 > 70 Gy) should be delivered for definitive intent, and concurrent chemotherapy may benefit individual cases.

## Simple Summary

Roughly 25% of non-small cell lung cancer (NSCLC) patients subsequently experience isolated locoregional recurrence following definitive radiation therapy (RT). For these scenarios, thoracic reirradiation (re-RT) has become an increasingly common consideration, with improved feasibility due to technological advancements that have made it safer to deliver high doses of RT in the retreatment setting. Proton beam therapy (PBT) is an attractive choice for re-RT due to its ability to spare radiation to adjacent previously treated normal tissues. Here, we evaluate outcomes with PBT for the definitive re-RT of recurrent NSCLC within the previously irradiated thorax.

## 1. Introduction

Lung cancer has been among the most commonly diagnosed cancers in the United States for decades [1] and the leading cause of cancer-related mortality worldwide [2], with non-small cell lung cancer (NSCLC) accounting for 80% of new diagnoses [3]. Earlier detection and advancements in treatment options have contributed to longer life expectancies among patients diagnosed with lung cancer [4,5]. Still, approximately 30% of lung cancers present in advanced stages and are more suitable for definitive radiation therapy in lieu of surgical resection [6]. Studies focused more specifically on the use of proton beam therapy (PBT) with concurrent chemotherapy report favorable treatment outcomes, describing it as a feasible and safe option for inoperable locally advanced NSCLC patients being treated with definitive intent [7,8,9,10]. Yet, treatment failure is not uncommon with the long-term follow-up of prospective trials indicating that 20–30% of patients can experience isolated locoregional recurrence (LRR) following definitive chemoradiation (chemo-RT) for Stage III disease [11,12].

For isolated locoregional recurrences, systemic therapy alone—along the lines of palliative management—portends a poor prognosis with response rates around 30% and a median survival of less than a year [13,14]. Reirradiation has emerged as an alternative treatment option in response to the previously described suboptimal outcomes and is cautiously utilized with increasing frequency [15]. A review of findings from previously published studies reported that re-RT with external beam radiation therapy (EBRT) to the chest in locally recurrent NSCLC patients was well tolerated with acceptable toxicity incidence [16]. Treatment planning for these individuals is complicated due to the high likelihood of dose overlap with previously irradiated thoracic structures.

However, advancements in particle therapy have made it possible to maintain radiation efficacy while decreasing patient safety risk, thereby enhancing the therapeutic ratio for the retreatment of overlapping areas. Proton beam radiotherapy is characterized by its lower exit dose which makes it potentially well suited for delivering appropriate prescription doses for definitive local control while mitigating excess toxicity risk [17,18] by sparing normal tissue surrounding the radiation treatment target [19]. The proximity of lung cancer treatment targets to critical structures including the esophagus, heart, normal lung, and spinal cord could make the use of PBT particularly advantageous for the reirradiation of new or recurrent lung cancers within or adjacent to previously irradiated areas.

## 2. Materials and Methods

### 2.1. Patients

Following the institutional review board protocol approval of our retrospective cohort study, patients who received salvage reirradiation with proton beam therapy for NSCLC within or adjacent to a prior definitive radiation therapy (RT) field were identified via an institutionally approved database. Patients all had biopsy confirmation to establish initial diagnosis prior to the first thoracic RT and were treated at a single institution between 2012 and 2021. All patients underwent multidisciplinary evaluation and radiographic restaging prior to subsequent courses of thoracic RT. Relevant information including demographics, medical history, smoking status, staging work-up, and oncologic treatment history (including systemic therapy) were extracted from the electronic health record.

### 2.2. Radiation Treatment and Planning

For treatment planning, positron emission tomography (PET) with computed tomography (CT) fusion (PET/CT fusion) was obtained prior to re-RT and used for contouring the target volumes for every retreatment case. PET/CT fusion was used both for appropriate patient selection (evaluating other sites of disease prior to aggressive management), as well as for accurate and precise treatment tailored to active high-risk disease (while minimizing unnecessary RT and toxicity risks for uninvolved tissue in the retreatment setting). All thoracic treatment plans accounted for respiratory motion via four-dimensional computed tomography (4D-CT) simulation. The internal gross tumor volume (iGTV) target was contoured upon spatial evaluation across every phase of the breathing cycle. Furthermore, 4D verification simulations occurred during the first week of every treatment course and every 2–3 weeks thereafter depending on course length to ensure the maintenance of intended dose coverage and facilitate adaptive planning as needed.

Radiation therapy technique, modality, prescription, and delivery dates were recorded. In an effort to exclude palliative treatment courses from analysis, “salvage reirradiation” was defined as a minimum BED10 > 50 Gy (corresponding to the lowest reasonable prescriptions of 45 Gy/15–30 fxs [BED10 = 52–59 Gy], though these accounted for a minority of courses as reflected in the Results.

Complete treatment records were available for all subjects, including a review of each treatment plan with dosimetric variables collected for relevant thoracic organs-at-risk. Composite plans were also generated for each patient following every thoracic re-RT course, documenting the cumulative dose imparted from all RT courses summed to date, based off dose deformation onto the most recent CT simulation planning scan. While all RT courses were delivered to the thorax, direct overlap between courses was noted for the treatment of the same site and for cases with an intersection of ≥50% isodose lines among different treatment plans.

### 2.3. Follow-Up

After the completion of PBT re-RT, patients continued surveillance follow-up with clinic visits every 2–3 months timed with repeat axial imaging (either CT or PET/CT scans) as per our standard institutional practice. Charts were reviewed for treatment-related toxicities following the last course of thoracic re-RT and scored in accordance with the National Cancer Institute Common Terminology Criteria for Adverse Events (CTCAE) version 5.0.

### 2.4. Statistical Analysis

Primary endpoints were overall survival (OS), progression-free survival (PFS), and toxicity, with actuarial estimates calculated via the Kaplan–Meier method from the last treatment date of re-RT to the date of last follow-up, death, and/or disease progression (as first noted on imaging). Cox proportional hazards modeling was used to assess for associations with time-dependent endpoints, while logistic regression was employed to evaluate associations with toxicity. Mann–Whitney U tests were used to compare dosimetric variables.

## 3. Results

### 3.1. Patient Characteristics

Patient, tumor, and treatment characteristics are summarized in greater detail in Table 1. Patients received IMPT (32%) or passive scatter (68%) for the re-RT of recurrent NSCLC to a median BED10 of 79 Gy (IQR 71–84 Gy), corresponding to a definitive prescription of 66 Gy/33 fxs (IQR 52.5 Gy/15 fxs to 70 Gy/35 fxs). More than half (56%) received cytotoxic chemotherapy with platinum doublet (most commonly carboplatin/paclitaxel) in close temporal proximity to proton re-RT: thirty-three (50%) concurrently and four (6%) as an induction (pre-RT).

PBT retreatment occurred at a median time of 1.4 years (IQR 0.9–2.1) from prior thoracic RT, with 18% of patients receiving two–three definitive courses previously, whereas the majority (82%) received a single course of prior RT. A total of 37 patients (56%) had received photon SBRT only, while 29 (44%) had received conventionally fractionated courses including 16 IMRT/VMAT and 13 PBT, with 4 of these longer course patients also having a prior history of SBRT. On analysis, however, no significant differences were noted with respect to OS (*p* = 0.589) or PFS (*p* = 0.894) following proton re-RT based on prior treatment modality (i.e., previous SBRT courses only versus other modalities). Of the 79 prior RT courses, 49 were delivered via stereotactic techniques (with BED10 > 100 Gy), most commonly to 50 Gy/4 fxs or 70 Gy/10 fxs for more centrally located disease. The remaining 30 entailed fractionated courses to a median BED10 of 80 Gy (IQR 71–89 Gy), corresponding to prescriptions of 66 Gy/30 fxs (IQR 52.5 Gy/15 fxs to 74 Gy/37 fxs), of which 13 were also delivered previously with PBT.

### 3.2. Clinical Outcomes

At a follow-up time of 14.2 months (IQR 7.4–30.7), the median OS and PFS were 15 months (95% CI: 13–17) and 12.5 months (95% CI: 9.6–15.4), respectively. Actuarial OS at 1 year and 2 years was 60 (±6)% and 31 (±6)%, corresponding to PFS estimates of 54 (±6)% and 25 (±5)% for the same timepoints. Interestingly, despite IMPT starting at our center in 2014, while passive scatter began in 2010, there was no significant difference in terms of the follow-up time between the techniques at a median 16 months versus 14 months, respectively (*p* = 0.271). Altogether, 31 patients (47%) ultimately demonstrated progression, including isolated local (in-field) failures in 10 (15%), distant metastases in 14 (21%), and combined recurrence at both sites in 7 (11%).

On univariate analysis, OS was adversely associated with older age (>70 years) [HR 1.90, *p* = 0.037] and a greater number of prior RT courses (HR 2.03, *p* = 0.040), while concurrent chemotherapy (HR 0.56, *p* = 0.04) and a higher RT dose (BED10 > 70 Gy) [HR 0.46, *p* = 0.016] were associated with improved survival. However, on multivariable analysis, only older age (HR 2.08, 95% CI: 1.13–38.5, *p* = 0.020) and a higher RT dose (HR 0.41, 95% CI: 0.22–0.79, *p* = 0.007) remained associated, with chemotherapy losing significance.

For PFS, univariate analysis found a greater number of prior RT courses was associated with worse PFS (HR 2.06, *p* = 0.033), and trends were observed for treatment site overlap (HR 1.47, *p* = 0.146) as well as the passive scatter PBT delivery technique (HR 1.71, *p* = 0.075). Chemotherapy (HR 0.53, *p* = 0.020) and a higher RT dose (HR 0.43, *p* = 0.004) were again protective. On multivariable analysis, a higher RT dose (HR 0.37, 95% CI 0.20–0.68, *p* = 0.001), concurrent chemotherapy (HR 0.48, 95% CI 0.28–0.81, *p* = 0.007), and treatment site overlap (HR 1.78, 95% CI 1.05–3.02, *p* = 0.031) were identified as significant factors.

**Figure 1:**
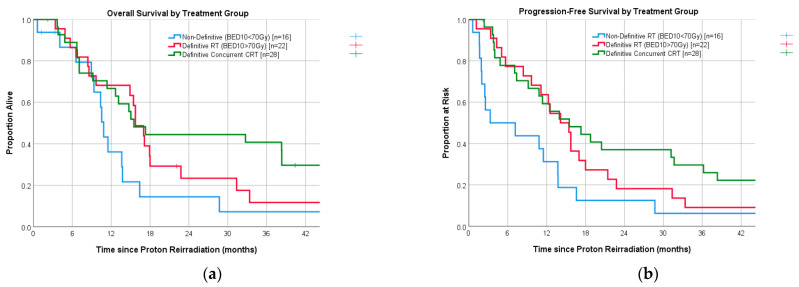
(**a**) Median OS for CRT, DRT alone, and LDRT was 15.5 (95% CI: 11.1–19.9), 15.7 (95% CI: 13.5–18), and 10.8 months (95% CI: 10–11.7) [log-rank *p* = 0.023]. Corresponding 1-year/2-year estimates for the three groups were 67 (±9)/44 (±10)%; 68 (±10)/23 (±10)%; and 36 (±13)/14 (±9)%. (**b**) Median PFS for CRT, DRT alone, and LDRT was 15.5 (95% CI: 7.3–23.7), 14.1 (95% CI: 10.9–17.3), and 3.3 months (95% CI: 0–12.3) [log-rank, *p* = 0.006]. The 1-year/2-year rates were 59 (±10)/37 (±9)%; 64 (±10)/18 (±8)%; and 31 (±12)/12.5 (±8)%.

The observed associations with chemotherapy and a higher RT dose with both OS and PFS are further evaluated in Figure 1, with a visual representation of the differences seen among three treatment groups: a higher RT dose with concurrent chemotherapy (*n* = 28), a higher RT dose alone without chemotherapy (*n* = 22), and a lower RT dose (BED10 < 70 Gy) [*n* = 16]. No significant associations were identified with respect to OS or PFS and comorbidities, gender, smoking history, initial stage at first presentation, surgical history, central treatment site, prior RT technique, and time interval since diagnosis or the last RT course.

### 3.3. Toxicity and Dosimetric Variables

There were a total of thirty-three G2+ adverse events in twenty-one patients (32%), including nine G3+ (one G4) events in seven patients (11%). All toxicities were noted to have occurred within 9 months post-RT completion. There were no Grade 5 events and no observed cardiac or neurologic toxicities. Esophageal toxicity was the most common of these, occurring in 18 patients (27%), with eighteen events of acute esophagitis (sixteen G2; two G3) noted by end-of-treatment, and one G3 esophageal stricture noted during post-RT follow-up. Acute dermatitis (two G2, one G3) was also noted in three patients by end-of-treatment.

Pulmonary toxicities also occurred in eight patients (12%): six pneumonitis events were observed (three G2, three G3), all within 3 months of treatment completion. Other pulmonary toxicities in three patients (4.5%) included three bronchial stenoses/strictures (two G2, one G3) and one G2 bronchopleural fistula observed at a median time of 5 months post-RT. There was one episode of G4 acute bronchopulmonary hemorrhage after treatment completion. This patient’s re-RT course took place within 7 months of his preceding RT and entailed the overlap of a central treatment site, corresponding with cumulative Dmax (EQD2) to the proximal bronchial tree and great mediastinal vessels of 140 Gy.

Cumulative dosimetric variables of composite treatment plans for the different subgroups are presented in Table 2, after conversion to the equivalent dose in 2 Gy fractions (EQD2). For re-RT cases with central site overlap, Dmax to the esophagus (median 87 Gy [IQR 77–90]), mediastinal vessels (median 120 Gy [IQR 110–138]), and proximal bronchial tree (median 120 Gy [IQR 110–138]) as well as the esophageal mean dose (median 21 Gy [IQR 17–33]) were significantly higher than for all other cases (*p* ≤ 0.001 for all variables). However, the only dosimetric factor associated with toxicity was the esophageal mean with Grade 2+ esophageal AEs (*p* = 0.018). Otherwise, no significant patterns were identified with respect to other AEs, with some G3+ toxicities occurring at normal expected constraint ranges while other cases were treated to much higher cumulative doses without experiencing toxicity.

With respect to clinical factors, the only significant factor associated with G2+ pulmonary toxicity was a greater number of prior RT courses (OR 6.3, 95% CI 1.3–30.1, *p* = 0.022). As for G2+ esophageal toxicity, multivariable analysis demonstrated significant associations with central location (OR 5.3, 95% CI 1.2–22.7, *p* = 0.025) and concurrent chemotherapy (OR 12.1, 95% CI 2.5–58.8, *p* = 0.002), while a longer time interval (>1 year) since a prior RT course was protective (OR 0.21, 95% CI 0.05–0.82, *p* = 0.025). No associations were identified for the toxicity endpoint (neither pulmonary nor esophageal) with respect to the PBT technique, re-RT prescription dose, medical comorbidities, surgical history, or smoking history.

## 4. Discussion

Here, we report outcomes and toxicity in patients treated with salvage PBT for recurrent NSCLC within the previously irradiated thorax, contributing to the limited institutional experiences of thoracic reirradiation for recurrent NSCLC and even fewer specifically focused on retreatment with PBT. The consensus among prior publications is that reirradiation with PBT is tolerable and safe but not without an acceptable risk of sometimes significant toxicities [20,21,22,23,24,25].

For our retrospective cohort, the median time between prior thoracic RT and salvage re-RT was 1.4 years, consistent with reirradiation guideline suggestions favoring a treatment break interval of >12 months between definitive courses. Notably, 56% percent of subjects received cytotoxic chemotherapy in close temporal proximity with proton re-RT, with better OS and PFS outcomes observed among the 50% of patients receiving chemotherapy concurrently, in accordance with other published reports [21,22,23,25]. However, this potential benefit should be balanced with the association between concurrent chemotherapy and increased risk for toxicities [23], notably Grade 2+ esophagitis identified in our study. It is our opinion that concurrent chemotherapy should be considered and reserved for isolated locoregional recurrences with nodal involvement in the absence of distant metastatic disease.

The incidence of any Grade 2+ toxicity was 32%, which is similar to what has been reported previously [20,21,24]. We found associations between Grade 2+ esophageal toxicity and central site overlap, concurrent chemotherapy, and a longer time interval from prior RT, while Grade 2+ pulmonary toxicity was associated with a greater number of prior thoracic RT courses delivered. Grade 3 events were observed among 11% of patients (including one Grade 4 bronchopulmonary hemorrhage), but no reliable dosimetric predictors were identified, likely due to the small overall number of high-grade adverse events. Other studies have reported a relationship between higher cumulative re-RT doses and esophageal toxicity [20,25]. Furthermore, previous studies have documented an increased risk for toxicity in the treatment of central tumors, especially those located near the proximal bronchial tree and esophagus [20,21,25]. While PBT allows for the more concentrated delivery of radiation to disease sites with a lower exit dose, the exposure of critical organs immediately adjacent to the treatment field is unavoidable particularly with robustness optimization for plan delivery. However, emerging treatment methods, like stereotactic body proton therapy (SBPT), provide promising alternative options for high-risk tumors, such as those with an ultracentral location and in-field recurrences [26].

Consistent with other proton reirradiation studies, our 2-year actuarial overall survival and progression-free survival rates of 31% and 25%, respectively, were indicative of relatively poor prognosis for all comers but with some long-term survivors. Previously identified factors which might be considered during patient selection for salvage re-RT include tumor volume, location, and relevant dosimetric parameters [21]; performance status (ECOG ≤ 1) and the ability to tolerate concurrent chemotherapy with definitive re-RT doses [22]; and the time elapsed between the initial radiation treatment and recurrence [27]. Proper patient selection is especially crucial in the high-risk scenario of reirradiation with overlapping fields and adhering to suggestions such as those put forth by several authors can help enhance the risk–benefit ratio. Our finding of an association between age > 70 and mortality may merely reflect the shorter life expectancy with older age, as no associations with toxicity endpoints were identified with respect to patient age.

In earlier studies regarding the use of photon-based external beam radiotherapy in the retreatment setting for locoregionally recurrent NSCLC, investigators concluded that reirradiation was safe, efficacious, and feasible for carefully selected patients [16]. Esophageal toxicity, mainly Grade 2 acute esophagitis, was the most common adverse event among our cohort, observed in 27% of patients. Previously, Okamoto et al., Wu et al., and Tada et al. reported somewhat lower rates of esophagitis (18% G2-3, 9% G1-2, and 16% G2, respectively) [28,29,30]. This might be partially owed to differences in tumor location and, consequently, the overlap of initial and subsequent RT treatment fields and the much less prevalent use of concurrent chemotherapy at the time of re-RT. Regarding pneumonitis, we report an incidence rate of 9% for G2-3 toxicity, lower than what has been previously reported in reirradiation studies with EBRT [28,29]. This difference could be reflective of the tissue-sparing capabilities of PBT compared to conventional RT modalities; however, the analysis of data from prospective trials would be necessary to ascertain an association. Toxicity rates documented in studies regarding the use of SBRT for reirradiation following initial treatment with EBRT varied but were similar to our rate of 32% ≥ Grade 2 toxicity [31], with Kelly et al. reporting a 33% Grade 3 toxicity rate, Reyngold et al. 23% G2-3 pulmonary toxicity, Trovo et al. with 23% Grade 2 pulmonary toxicity, and Parks et al. a 25.9% overall Grade 2 toxicity rate [32,33,34,35]. Studies with lower rates of ≥Grade 2 toxicity involved a majority of out-of-field recurrences [36], and a longer median interval between initial RT treatment and SBRT reirradiation [36,37,38]. Overall, rates of pulmonary toxicity, including pneumonitis, tended to be higher [32,33,34,35,36], while reported rates of esophagitis were generally lower [32,35]. Given the historical observation of pneumonitis as the most common adverse event associated with SBRT, this difference is not entirely unexpected. Moreover, the nature and location of tumors that are treated with SBRT compared to PBT, which can be applied to more centrally located tumors, might explain differences in the rates of esophagitis observed among our patient cohort as compared to those in SBRT re-RT studies.

Among our cohort, 31 patients (47%) ultimately demonstrated progression, including local (in-field) treatment failures noted in 17 (26%), of which only 10 (15%) represented isolated local recurrences in the absence of distant progression. The authors of previous PBT reirradiation studies have similarly reported high rates of LRR and distant metastasis [20,21,22,24], which is not unexpected for recurrences which are analogous to the presentation of Stage III-IV disease. Due to these high rates of progression, there remains an opportunity for further investigation into the potential role of targeted therapies and immunotherapy for the further improvement in prognoses among this recurrent population, as highlighted in previously published reports [39,40,41,42]. Two retrospective cohort studies found that patients treated with PBT re-RT and consolidation immunotherapy showed a survival benefit compared to those who were treated with re-RT alone [27,43]. Moreover, in a recently published prospective study focused on the use of adjuvant immunotherapy following PBT re-RT for NSCLC recurrence, acceptable PFS and favorable OS were reported [44].

The limitations of our study include its retrospective nature and heterogeneity in treatment history, including prior RT, re-RT prescriptions, and the use of systemic therapy. Additionally, no direct comparisons could be made between outcomes and toxicity reported here and those associated with other salvage radiation modalities. Further, we unfortunately lack access to internal data on previous IMRT trials which might have been considered prior to opting for PBT as these plans were not routinely processed, signed, and saved for future review. Thus, comparisons could not be made between plans to assess differences in how well PBT reduced a high dose in close proximity compared to photons, considering that protons lose some high-dose conformality in lower density lung tissue. Moreover, while we recognize that the LET distribution may differ between IMPT and passive scattering techniques, LET-based modeling is not currently routinely employed as part of our clinical practice and could thus not be taken into account during follow-up. Lastly, the fact that the majority of patients in our cohort were treated in a previous era facilitated a longer follow-up; however, this came at the cost of only a fraction receiving modern immunotherapy or targeted therapies for analysis in this report. However, this represents among the largest single-institution cohort of patients, with the added benefit of having composite radiation treatment plans available for analysis with cumulative doses imparted to thoracic organs-at-risk. Our work thus contributes to the growing body of literature aimed at evaluating outcomes, feasibility, and patient selection for aggressive salvage therapy using modern reirradiation techniques.

## 5. Conclusions

Thoracic reirradiation with proton beam therapy is feasible with acceptable toxicity and promising outcomes for select patients which may be further enhanced by concurrent chemotherapy for individual cases. Our data suggest that higher prescription doses (BED10 > 70 Gy) should be delivered for definitive intent whenever feasible. However, given the persistent risk of significant adverse events, additional research is warranted on establishing literature-supported dose constraints for the reirradiation setting.

## Figures and Tables

**Table 1:** Patient, tumor, and treatment characteristics (*n* = 66).

Characteristic	Total (*N* = 66)
Age at re-RT, Years	
Median (IQR)	73 (66–78)
Sex	
Female	35 (53%)
Male	31 (47%)
Smoking History	
Non-smoker	7 (11%)
Current or former smoker	59 (89%)
Median pack years (IQR)	44 (22–60)
Histology	
Adenocarcinoma	28 (42%)
Squamous cell carcinoma	30 (45%)
NSCLC NOS	8 (12%)
Medical History	
COPD/emphysema	40 (61%)
Surgical resection	18 (27%)
Cardiac disease	23 (35%)
Initial Stage (First Presentation)	
I	37 (56%)
II	9 (14%)
III	17 (26%)
IV (oligometastatic)	3 (5%)
Median Time Elapsed, Years (IQR)	
From initial diagnosis to PBT	2.0 (1.1–3.2)
From last RT to PBT re-RT	1.4 (0.9–2.1)
From PBT re-RT to latest follow-up	1.2 (0.6–2.6)
PBT re-RT Technique	
IMPT (pencil beam scanning)	21 (32%)
PSPT (passive scatter)	45 (68%)
Prior Radiation	
1 course	54 (82%)
≥2 courses	12 (18%)
Chemotherapy with PBT re-RT	
Induction	4 (6%)
Concurrent	33 (50%)
Treatment Sites (PBT re-RT)	
Mediastinum	40 (61%)
Hilar/perihilar	18 (27%)
Direct overlap (in-field)	30 (45%)
Central fields overlap	18 (27%)
Site of Progression following PBT re-RT	
Any	31 (47%)
Distant	14 (21%)
In-field (isolated local failure)	10 (15%)
Mixed (local and distant)	7 (11%)
Toxicities	
Grade 2	18 (27%)
Grade 3	6 (9%)
Grade 4	1 (1.5%)

Abbreviations: IQR, interquartile range; NSCLC, non-small cell lung cancer; NOS, not otherwise specified; COPD, chronic obstructive pulmonary disease; PBT, proton beam therapy; re-RT, reirradiation; RT, radiation therapy; IMPT, intensity-modulated proton therapy; PSPT, passively scattered proton therapy.

**Table 2:** Dosimetric variables associated with different subgroups, presented as median EQD2 values with corresponding ranges.

OAR Dosimetry (EQD2)	All Cases	Central Overlap	G2+ Pulmonary	G2+ Esophageal	Any Grade 3+
Lung [range] (IQR)					
Mean (Gy)	14 [3–34]	16 (11–19)	15 (11–19)	14 (12–18)	19 (13–20)
V20 Gy (%)	23 [2–50]	24 (17–32)	21 (15–35)	25 (20–30)	35 (23–37)
Heart [range] (IQR)					
Mean (Gy)	5 [0–38]	4 (2–11)	6 (3–21)	7 (4–13)	8 (5–21)
Dmax (Gy)	74 [2–155]	72 (58–91)	73 (68–96)	73 (71–94)	74 (72–98)
Esophagus [range] (IQR)					
Mean (Gy)	17 [1–58]	21 (17–33)	18 (13–30)	22 (17–30)	19 (16–32)
Dmax (Gy)	73 [5–127]	87 (77–90)	71 (67–84)	76 (70–85)	77 (67–85)
Proximal Bronchial Tree					
Dmax, Gy [range] (IQR)	90 [15–153]	120 (110–138)	98 (85–121)	107 (83–123)	100 (77–110)
Great Mediastinal Vessels			[2–50]		
Dmax, Gy [range] (IQR)	90 [15–153]	120 (110–138)	95 (85–121)	97 (81–110)	110 (107–135)

## Data Availability

The data presented in this study may be available upon request to the corresponding author.

## References

[1] Wingo P.A., Cardinez C.J., Landis S.H., Greenlee R.T., Ries L.A.G., Anderson R.N., Thun M.J. (2003). Long-Term Trends in Cancer Mortality in the United States, 1930–1998. Cancer.

[2] Siegel R.L., Giaquinto A.N., Jemal A. (2024). Cancer statistics, 2024. CA. Cancer J. Clin..

[3] Sankar V., Kothai R., Vanisr N. (2023). Lung Cancer—A Review. Int. J. Health Sci. Res..

[4] de Koning H.J., van der Aalst C.M., de Jong P.A., Scholten E.T., Nackaerts K., Heuvelmans M.A., Lammers J.-W.J., Weenink C., Yousaf-Khan U., Horeweg N. (2020). Reduced Lung-Cancer Mortality with Volume CT Screening in a Randomized Trial. N. Engl. J. Med..

[5] Howlader N., Forjaz G., Mooradian M.J., Meza R., Kong C.Y., Cronin K.A., Mariotto A.B., Lowy D.R., Feuer E.J. (2020). The Effect of Advances in Lung-Cancer Treatment on Population Mortality. N. Engl. J. Med..

[6] Hansen R.N., Zhang Y., Seal B., Ryan K., Yong C., Darilay A., Ramsey S.D. (2020). Long-Term Survival Trends in Patients with Unresectable Stage III Non-Small Cell Lung Cancer Receiving Chemotherapy and Radiation Therapy: A SEER Cancer Registry Analysis. BMC Cancer.

[7] Tatebe H., Harada H., Mori K., Iwata H., Akimoto T., Murakami M., Waki T., Ogino T., Nakamura M., Taguchi H. (2023). Clinical Results of Proton Beam Radiotherapy for Inoperable Stage III Non-Small Cell Lung Cancer: A Japanese National Registry Study. J. Radiat. Res..

[8] Nguyen Q.-N., Ly N.B., Komaki R., Levy L.B., Gomez D.R., Chang J.Y., Allen P.K., Mehran R.J., Lu C., Gillin M. (2015). lLong-Term Outcomes after Proton Therapy, with Concurrent Chemotherapy, for Stage II-III Inoperable Non-Small Cell Lung Cancer. Radiother. Oncol. J. Eur. Soc. Ther. Radiol. Oncol..

[9] Oshiro Y., Okumura T., Kurishima K., Homma S., Mizumoto M., Ishikawa H., Onizuka M., Sakai M., Goto Y., Hizawa N. (2014). High-Dose Concurrent Chemo–Proton Therapy for Stage III NSCLC: Preliminary Results of a Phase II Study. J. Radiat. Res..

[10] Chang J.Y., Verma V., Li M., Zhang W., Komaki R., Lu C., Allen P.K., Liao Z., Welsh J., Lin S.H. (2017). Proton Beam Radiotherapy and Concurrent Chemotherapy for Unresectable Stage III Non–Small Cell Lung Cancer: Final Results of a Phase 2 Study. JAMA Oncol..

[11] Spigel D.R., Faivre-Finn C., Gray J.E., Vicente D., Planchard D., Paz-Ares L., Vansteenkiste J.F., Garassino M.C., Hui R., Quantin X. (2022). Five-Year Survival Outcomes from the PACIFIC Trial: Durvalumab after Chemoradiotherapy in Stage III Non–Small-Cell Lung Cancer. J. Clin. Oncol..

[12] Bradley J.D., Hu C., Komaki R.R., Masters G.A., Blumenschein G.R., Schild S.E., Bogart J.A., Forster K.M., Magliocco A.M., Kavadi V.S. (2020). Long-Term Results of NRG Oncology RTOG 0617: Standard- Versus High-Dose Chemoradiotherapy with or without Cetuximab for Unresectable Stage III Non–Small-Cell Lung Cancer. J. Clin. Oncol..

[13] De Ruysscher D., Faivre-Finn C., Le Pechoux C., Peeters S., Belderbos J. (2014). High-Dose Re-Irradiation Following Radical Radiotherapy for Non-Small-Cell Lung Cancer. Lancet Oncol..

[14] Peters S., Adjei A.A., Gridelli C., Reck M., Kerr K., Felip E. (2012). Metastatic Non-Small-Cell Lung Cancer (NSCLC): ESMO Clinical Practice Guidelines for Diagnosis, Treatment and Follow-Up. Ann. Oncol..

[15] Paradis K.C., Mayo C., Owen D., Spratt D.E., Hearn J., Rosen B., Kashani R., Moran J., Tatro D.S., Beeler W. (2019). The Special Medical Physics Consult Process for Reirradiation Patients. Adv. Radiat. Oncol..

[16] Jeremić B., Videtic G.M.M. (2011). Chest Reirradiation with External Beam Radiotherapy for Locally Recurrent Non-Small-Cell Lung Cancer: A Review. Int. J. Radiat. Oncol..

[17] Socinski M.A., Morris D.E., Halle J.S., Moore D.T., Hensing T.A., Limentani S.A., Fraser R., Tynan M., Mears A., Rivera M.P. (2004). Induction and Concurrent Chemotherapy with High-Dose Thoracic Conformal Radiation Therapy in Unresectable Stage IIIA and IIIB Non–Small-Cell Lung Cancer: A Dose-Escalation Phase I Trial. J. Clin. Oncol..

[18] Patel N.V., Yu N.Y., Koroulakis A., Diwanji T., Sawant A., Sio T.T., Mohindra P. (2021). Proton Therapy for Thoracic Malignancies: A Review of Oncologic Outcomes. Expert Rev. Anticancer. Ther..

[19] Simone C.B., Rengan R. (2014). The Use of Proton Therapy in the Treatment of Lung Cancers. Cancer J..

[20] McAvoy S.A., Ciura K.T., Rineer J.M., Allen P.K., Liao Z., Chang J.Y., Palmer M.B., Cox J.D., Komaki R., Gomez D.R. (2013). Feasibility of Proton Beam Therapy for Reirradiation of Locoregionally Recurrent Non-Small Cell Lung Cancer. Radiother. Oncol..

[21] Chao H.-H., Berman A.T., Simone C.B., Ciunci C., Gabriel P., Lin H., Both S., Langer C., Lelionis K., Rengan R. (2017). Multi-Institutional Prospective Study of Reirradiation with Proton Beam Radiotherapy for Locoregionally Recurrent Non–Small Cell Lung Cancer. J. Thorac. Oncol..

[22] McAvoy S., Ciura K., Wei C., Rineer J., Liao Z., Chang J.Y., Palmer M.B., Cox J.D., Komaki R., Gomez D.R. (2014). Definitive Reirradiation for Locoregionally Recurrent Non-Small Cell Lung Cancer with Proton Beam Therapy or Intensity Modulated Radiation Therapy: Predictors of High-Grade Toxicity and Survival Outcomes. Int. J. Radiat. Oncol..

[23] Badiyan S.N., Rutenberg M.S., Hoppe B.S., Mohindra P., Larson G., Hartsell W.F., Tsai H., Zeng J., Rengan R., Glass E. (2019). Clinical Outcomes of Patients with Recurrent Lung Cancer Reirradiated with Proton Therapy on the Proton Collaborative Group and University of Florida Proton Therapy Institute Prospective Registry Studies. Pract. Radiat. Oncol..

[24] Shin H., Noh J.M., Pyo H., Ahn Y.C., Oh D. (2021). Salvage Proton Beam Therapy for Locoregional Recurrence of Non-Small Cell Lung Cancer. Radiat. Oncol. J..

[25] Yang K., Suh Y.-G., Shin H., Pyo H., Moon S.H., Ahn Y.C., Oh D., Chung E., Jo K., Noh J.M. (2022). Toxicity of Proton Therapy versus Photon Therapy on Salvage Re-Irradiation for Non-Small Cell Lung Cancer. Life Basel Switz..

[26] McMillan M.T., Shepherd A.F., Kang M., Lin L., Shaverdian N., Wu A.J., Gelblum D.Y., Ohri N., Lazarev S., Xu L. (2023). Safety and Efficacy of Stereotactic Body Proton Therapy for High-Risk Lung Tumors. J. Radiosurg. SBRT.

[27] Janopaul-Naylor J.R., Cao Y., McCall N.S., Switchenko J.M., Tian S., Chen H., Stokes W.A., Kesarwala A.H., McDonald M.W., Shelton J.W. (2022). Definitive Intensity Modulated Proton Re-Irradiation for Lung Cancer in the Immunotherapy Era. Front. Oncol..

[28] Okamoto Y., Murakami M., Yoden E., Sasaki R., Okuno Y., Nakajima T., Kuroda Y. (2002). Reirradiation for Locally Recurrent Lung Cancer Previously Treated with Radiation Therapy. Int. J. Radiat. Oncol..

[29] Wu K.-L., Jiang G.-L., Qian H., Wang L.-J., Yang H.-J., Fu X.-L., Zhao S. (2003). Three-Dimensional Conformal Radiotherapy for Locoregionally Recurrent Lung Carcinoma after External Beam Irradiation: A Prospective Phase I–II Clinical Trial. Int. J. Radiat. Oncol..

[30] Tada T., Fukuda H., Matsui K., Hirashima T., Hosono M., Takada Y., Inoue Y. (2005). Non-Small-Cell Lung Cancer: Reirradiation for Loco-Regional Relapse Previously Treated with Radiation Therapy. Int. J. Clin. Oncol..

[31] Milano M.T., Mihai A., Kong F.-M. (2018). (Spring) Review of Thoracic Reirradiation with Stereotactic Body Radiation Therapy. Pract. Radiat. Oncol..

[32] Kelly P., Balter P.A., Rebueno N., Sharp H.J., Liao Z., Komaki R., Chang J.Y. (2010). Stereotactic Body Radiation Therapy for Patients with Lung Cancer Previously Treated with Thoracic Radiation. Int. J. Radiat. Oncol..

[33] Reyngold M., Wu A.J., McLane A., Zhang Z., Hsu M., Stein N.F., Zhou Y., Ho A.Y., Rosenzweig K.E., Yorke E.D. (2013). Toxicity and Outcomes of Thoracic Re-Irradiation Using Stereotactic Body Radiation Therapy (SBRT). Radiat. Oncol..

[34] Trovo M., Minatel E., Durofil E., Polesel J., Avanzo M., Baresic T., Bearz A., Del Conte A., Franchin G., Gobitti C. (2014). Stereotactic Body Radiation Therapy for Re-irradiation of Persistent or Recurrent Non-Small Cell Lung Cancer. Int. J. Radiat. Oncol..

[35] Parks J., Kloecker G., Woo S., Dunlap N.E. (2016). Stereotactic Body Radiation Therapy as Salvage for Intrathoracic Recurrence in Patients with Previously Irradiated Locally Advanced Non–Small Cell Lung Cancer. Am. J. Clin. Oncol..

[36] Liu H., Zhang X., Vinogradskiy Y.Y., Swisher S.G., Komaki R., Chang J.Y. (2012). Predicting Radiation Pneumonitis after Stereotactic Ablative Radiation Therapy in Patients Previously Treated with Conventional Thoracic Radiation Therapy. Int. J. Radiat. Oncol..

[37] Owen D., Olivier K.R., Song L., Mayo C.S., Miller R.C., Nelson K., Bauer H., Brown P.D., Park S.S., Ma D.J. (2015). Safety and Tolerability of SBRT after High-Dose External Beam Radiation to the Lung. Front. Oncol..

[38] Repka M.C., Aghdam N., Kataria S.K., Campbell L., Suy S., Collins S.P., Anderson E., Lischalk J.W., Collins B.T. (2017). Five-Fraction SBRT for Ultra-Central NSCLC in-Field Recurrences Following High-Dose Conventional Radiation. Radiat. Oncol..

[39] Jabbour S.K., Berman A.T., Simone C.B. (2017). Integrating Immunotherapy into Chemoradiation Regimens for Medically Inoperable Locally Advanced Non-Small Cell Lung Cancer. Transl. Lung Cancer Res..

[40] Simone C.B., Burri S.H., Heinzerling J.H. (2015). Novel Radiotherapy Approaches for Lung Cancer: Combining Radiation Therapy with Targeted and Immunotherapies. Transl. Lung Cancer Res..

[41] Kalbasi A., June C.H., Haas N., Vapiwala N. (2013). Radiation and Immunotherapy: A synergistic Combination. J. Clin. Investig..

[42] Kang J., Demaria S., Formenti S. (2016). Current Clinical Trials Testing the Combination of Immunotherapy with Radiotherapy. J. Immunother. Cancer.

[43] Grambozov B., Wass R., Stana M., Gerum S., Karner J., Fastner G., Studnicka M., Sedlmayer F., Zehentmayr F. (2021). Impact of Reirradiation, Chemotherapy, and Immunotherapy on Survival of Patients with Recurrent Lung Cancer: A single-Center Retrospective Analysis. Thorac. Cancer.

[44] Yegya-Raman N., Berman A.T., Ciunci C.A., Friedes C., Berlin E., Iocolano M., Wang X., Lai C., Levin W.P., Cengel K.A. (2024). Phase 2 Trial of Consolidation Pembrolizumab after Proton Reirradiation for Thoracic Recurrences of Non-Small Cell Lung Cancer. Int. J. Radiat. Oncol. Biol. Phys..

